# Profile of circulating microRNAs in myalgic encephalomyelitis and their relation to symptom severity, and disease pathophysiology

**DOI:** 10.1038/s41598-020-76438-y

**Published:** 2020-11-12

**Authors:** Evguenia Nepotchatykh, Wesam Elremaly, Iurie Caraus, Christian Godbout, Corinne Leveau, Lynda Chalder, Catherine Beaudin, Emi Kanamaru, Renata Kosovskaia, Shawn Lauzon, Yanick Maillet, Anita Franco, Viorica Lascau-Coman, Saadallah Bouhanik, Yaned Patricia Gaitan, Dawei Li, Alain Moreau

**Affiliations:** 1 Viscogliosi Laboratory in Molecular Genetics of Musculoskeletal Diseases, Office 2.17.027, Sainte-Justine University Hospital Research Center, 3175 Cote-Ste-Catherine Road, Montreal, QC H3T 1C5 Canada; 2 Molecular Biology PhD Program, Faculty of Graduate and Postdoctoral Studies, Université de Montréal, 2900 Edouard Montpetit Blvd, Montreal, QC H3T 1J4 Canada; 3 Department of Biochemistry and Molecular Medicine, Faculty of Medicine, Université de Montréal, 2900 Edouard Montpetit Blvd, Montreal, QC H3T 1J4 Canada; 4 Patient-Partner, ICanCME Research Network, Sainte-Justine University Hospital Research Center, 3175 Cote-Ste-Catherine Road, Montreal, QC H3T 1C5 Canada; 5 Department of Microbiology and Molecular Genetics, University of Vermont, 95 Carrigan Drive, Burlington, VT 05405 USA; 6 Department of Stomatology, Faculty of Dentistry, Université de Montréal, 2900 Edouard Montpetit Blvd, Montreal, QC H3T 1J4 Canada

**Keywords:** Biochemistry, Computational biology and bioinformatics, Molecular biology, Biomarkers, Diseases, Medical research, Molecular medicine, Pathogenesis

## Abstract

Myalgic encephalomyelitis/chronic fatigue syndrome (ME/CFS) is a complex chronic disease, rooted in multi-system dysfunctions characterized by unexplained debilitating fatigue. Post-exertional malaise (PEM), defined as the exacerbation of the patient's symptoms following minimal physical or mental stress, is a hallmark of ME/CFS. While multiple case definitions exist, there is currently no well-established biomarkers or laboratory tests to diagnose ME/CFS. Our study aimed to investigate circulating microRNA expression in severely ill ME/CFS patients before and after an innovative stress challenge that stimulates PEM. Our findings highlight the differential expression of eleven microRNAs associated with a physiological response to PEM. The present study uncovers specific microRNA expression signatures associated with ME/CFS in response to PEM induction and reports microRNA expression patterns associated to specific symptom severities. The identification of distinctive microRNA expression signatures for ME/CFS through a provocation challenge is essential for the elucidation of the ME/CFS pathophysiology, and lead to accurate diagnoses, prevention measures, and effective treatment options.

## Introduction

Myalgic Encephalomyelitis/Chronic fatigue syndrome (ME/CFS) is a multi-system complex chronic disease of unknown etiology^1^. It afflicts approximately 600,000 Canadians and 2.5 million people in the United States. While multiple case definitions for ME/CFS exist, the Canadian Consensus Criteria (CCC 2003) focus on the most specific features of the disease^2^. These symptoms must include persistent fatigue, post-exertional malaise (PEM), sleep disturbances, localized or diffuse muscle pain, and another five out of 13 symptoms^2^ that must last for a minimum of six months. PEM is a hallmark of ME/CFS (among all symptoms) and is defined as the exacerbation of the patient's symptoms following minimal physical or mental stress. ME/CFS symptoms may also include postural orthostatic tachycardia syndrome (POTS), sound and light hypersensitivity, brain fog, and cognitive impairment^3^. Currently, there are no validated diagnostic biomarkers associated with ME/CFS. Physicians must diagnose ME/CFS through a clinical assessment to exclude other diseases with similar symptoms. Therefore, the discovery of specific biological biomarkers is essential to obtain accurate diagnoses, a better understanding of ME/CFS pathophysiology, and targeted treatments through precision medicine^4^.

MicroRNAs (miRNAs) are a class of small non-coding RNAs that regulate gene expression at the post-translational and/or post-transcriptional level by targeting mRNAs. MiRNAs have various roles in different biological processes, such as metabolism, cell survival and differentiation^5,6^. The dysregulation in the expression of miRNAs is involved in many diseases, including cancer progression^7,8^ and neurodegenerative diseases^9–12^. Previous studies revealed that differentially expressed miRNAs were associated with ME/CFS^13–15^. Detection of miRNA signatures that are indicative of the molecular mechanisms underlying specific ME/CFS core symptoms may allow biological insights into differentiating severe cases from mild forms of ME/CFS, giving clues to ME/CFS development and its pathophysiology. In the present study, we performed extensive profiling of circulating miRNAs on plasma samples of patients with severe ME/CFS (housebound), both at baseline and in response to the application of a post-exertional stress challenge. This unique experimental design led us to determine distinct molecular footprints of ME/CFS by comparing the differential expression plasma miRNA levels before and after 90 min of stimulation, which induced PEM, compared to age- and sex-matched controls. With implementation of a machine learning algorithm (i.e., Random Forest), we validated eleven microRNAs (hsa-miR-28-5p, hsa-miR-29a-3p, hsa-miR-127-3p, hsa-miR-140-5p, hsa-miR-150-5p, hsa-miR-181b-5p, hsa-miR-374b-5p, hsa-miR-486-5p, hsa-miR-3620-3p, hsa-miR-4433a-5p, and hsa-miR-6819-3p), the first diagnostic panel of its kind. Differential expression of these eleven circulating miRNAs led to the identification of four ME/CFS clusters with distinct miRNA profiles and specific symptom severities.

## Results

### Clinical and demographic characteristics of participants

**Figure 1:**
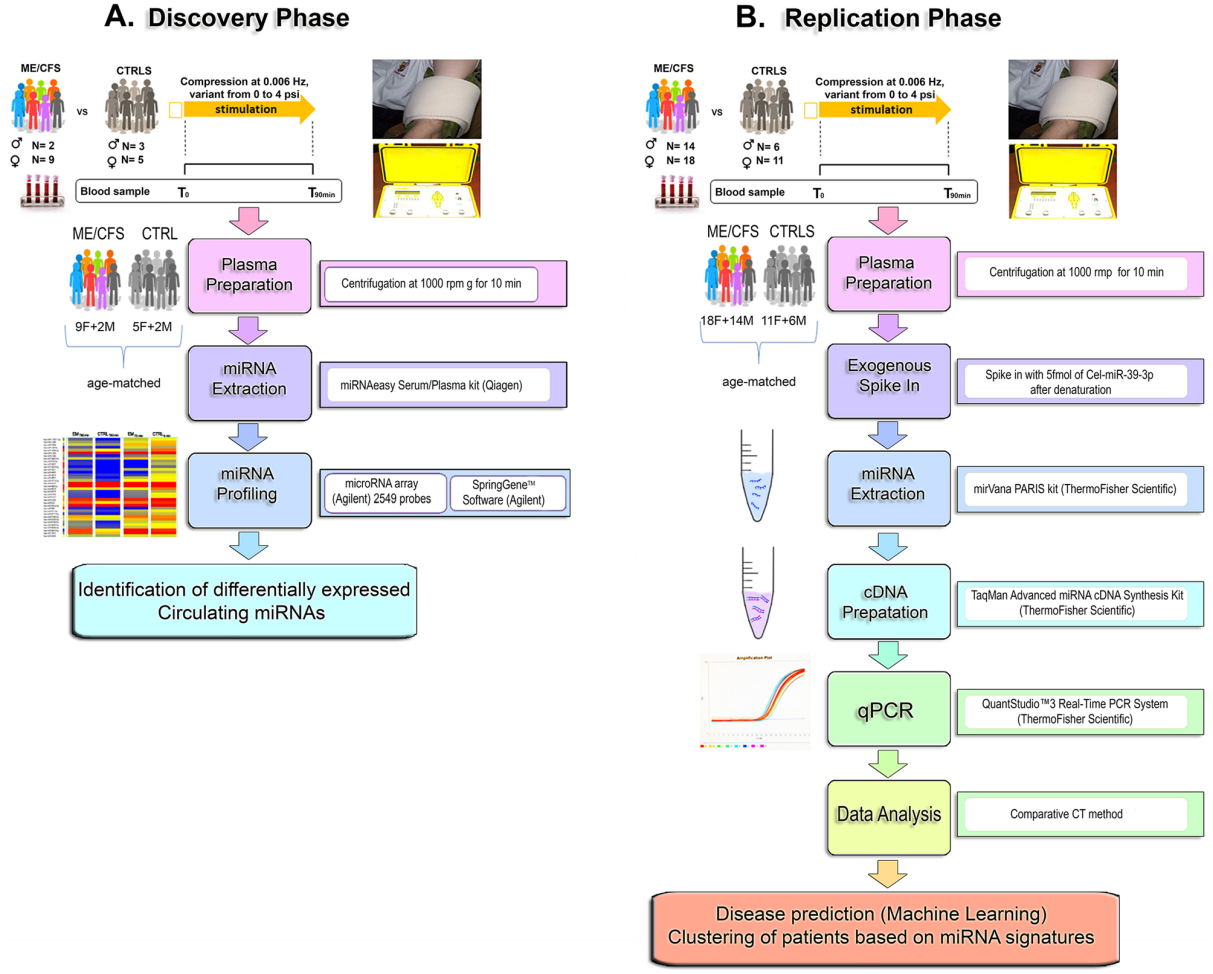
The experimental study design. Abbreviations: ME/CFS (encephalomyelitis/chronic fatigue syndrome), CTRLS (healthy matched controls), T0 (at baseline), T90 (after stress-test).

As per our study design, two blood samples were obtained from each participant, one at baseline and a second one after 90 min of stimulation involving the application of a post-exertional stress challenge (Fig. 1). For the discovery cohort, thirty-eight plasma samples (Table 1) were obtained from 11 ME/CFS patients (9 women and 2 men) and 8 matched healthy controls (5 women and 3 men). For the replication cohort, ninety-eight plasma samples (Table 1) were obtained from 32 ME/CFS patients (18 women and 14 men) and 17 matched controls (11 women and 6 men). For both cohorts, no significant differences in age, sex, and body mass index (BMI) were observed between ME/CFS patients and matched controls. There was no significant difference regarding illness duration between both ME/CFS cohorts (Table 1). All participants completed three self-reported questionnaires, Short Form 36-Item Health Survey (SF-36), Multidimensional Fatigue Inventory-20 (MFI-20) and DePaul Symptom Questionnaire (DSQ). As expected, significant differences in all health scores were observed between the ME/CFS groups and matched healthy controls (Table 1).

**Table 1:** Clinical and demographic characteristics of participants.

	Discovery cohort		Replication cohort	
	ME/CFS n = 11	CTRLs n = 8	ME/CFS n = 32	CTRLs n = 17
Age (years)	58 ± 2.3	58 ± 4	49.2 ± 2.1	49.8 ± 2.2
Body mass index (BMI) (kg/m^2^)	23.1 ± 1.2	24.0 ± 1.4	25.1 ± 0.8	25.5 ± 1.4
Sex (male/female)	2/9	3/5	14/18	6/11
Illness duration (years)	17 ± 2.0	N/A	14.6 ± 2.1	N/A
**36-Item Short-Form Health Survey (SF-36) Scores**				
Physical score	35 ± 7.1***	89 ± 5.0	35 ± 2.8***	91 ± 1.6
Mental score	46 ± 6.6***	86 ± 0.8	49 ± 3.5***	87 ± 2.2
**Multidimensional Fatigue Inventory-20 (MFI) Scores**				
General fatigue	19 ± 1***	7 ± 0.7	18 ± 0.5***	7 ± 0.7
Physical fatigue	18 ± 1.4***	6 ± 2.5	17 ± 0.6***	6 ± 0.7
Reduced activity	16 ± 1.1***	6 ± 3.1	15 ± 0.8***	6 ± 0.6
Reduced motivation	11 ± 0.8***	6 ± 1.9	11 ± 0.7***	5 ± 0.4
Mental fatigue	16 ± 1.4***	6 ± 4	15 ± 0.6***	6 ± 0.5
**DePaul Symptom Questionnaire (DSQ) Scores**				
Neuroendocrine, Autonomic and Immune Dysfunction score	46 ± 5.6***	7 ± 2.2	36 ± 2.8***	5 ± 0.6
Cognitive Dysfunction score	66 ± 6.2***	9 ± 3.1	55 ± 3.8***	5 ± 1.7
Post-exertional malaise (PEM) score	71 ± 6.6***	9 ± 1.9	67 ± 3.9***	8 ± 1.2
Sleep Disturbance score	43 ± 4.7***	12 ± 4	48 ± 3.1***	19 ± 1.3

Values for the different SF-36, MFI-20 and DSQ categories are described as scores. All data are represented as mean ± standard error of the mean.

2-Tailed Student T-test comparing ME/CFS patients and healthy controls were performed and were considered significant. *P value < 0.05, **P value < 0.01, ***P value < 0.001.

### Development of a standardized post-exertional stress challenge

PEM is a hallmark symptom differentiating ME/CFS from other related conditions. To reproduce PEM safely in participants with ME/CFS, we introduced a post-exertional stress challenge using a therapeutic massager device. Blood samples were taken at baseline (before stimulation) and after 90 min of stimulation from each participant to evaluate the changes in the miRNA expression profile in response to this mechanical stimulation. All participants were interviewed seven days afterwards to determine whether our post-exertional stress challenge induced or exacerbated symptoms associated with PEM, given that PEM development is highly variable from one individual to another. All ME/CFS subjects reported PEM symptoms, while none were reported by controls. Profound fatigue, headache, muscle pain, sleep disturbances and flu-like symptoms were the most frequently reported symptoms following the application of our post-exertional stress challenge (Supplementary Table S1). This innovative method presents several advantages over classical approaches measuring circulating miRNAs only at baseline without any challenge. First, this method allows for each participant to be their own experimental control given that changes in circulating miRNA profiles (or any other biomarkers) in response to our post-exertional stress challenge are more likely revealing disease-specific markers and reducing the confounding influence of other factors like current medication, illness duration, age, sex and even the presence of certain comorbidities. Secondly, our method has the merit to be portable, and cost-effective. It can allow the testing of individuals severely affected by ME/CFS (e.g. housebound) who rarely participate in clinical studies. Finally, the short period of stimulation (only 90 min) allows more rapid and direct measurement of immediate molecular changes occurring in response to PEM, contrasting with other approaches involving an exercise challenge over a one or two-day period^16–18^.

### Identification of individual miRNAs associated with ME/CFS and PEM

We used our discovery cohort in combination with the Agilent expression array-Human miRNA 8 × 60 K chips, for the identification of candidate circulating miRNAs differentially expressed in ME/CFS patients compared to healthy controls at baseline and/or after the post-exertional stress challenge. Seventeen miRNAs were identified as differently expressed after applying normalization steps using the GeneSpring software (Table 2). We found that at baseline the expression of hsa-miR-29a-3p, hsa-miR-150-5p, hsa-miR-181b-5p was elevated [highest fold-change (FC) =  + 2.86 and *P* < 0.05] and that of hsa-miR-4433a-5p and hsa-miR-6819-3p was reduced (− 6.46 FC, *P* < 0.001 and − 11.13 FC, *P* < 0.001 respectively) in the ME/CFS group compared to healthy controls. After 90 min of stimulation, the expression levels of hsa-miR-127-3p, hsa-miR-140-5p, hsa-miR-150-5p, hsa-miR-374b-5p, hsa-miR-5581-5p, hsa-miR-6076, hsa-miR-6717-5p, hsa-miR-6875-5p, increased and those of hsa-miR-486-5p, hsa-miR-3620-3p and hsa-miR-6507-3p decreased in the ME/CFS group compared to controls (Table 2). Furthermore, a comparison of miRNA expression levels at baseline versus after stimulation in ME/CFS group revealed additional changes with significant elevation in the expression of hsa-miR-28-5p, hsa-miR-29a-3p, hsa-miR-140-5p and hsa-miR-374b-5p, hsa-miR-6875-5p and decreased expression of hsa-miR-486-5p and hsa-miR-6800-3p. A similar comparison in the control group revealed an increased expression of hsa-miR-3620-3p and hsa-miR-6507-3p.

**Table 2:** Top 17 candidate miRNAs identified in microarray analysis deregulated between ME/CFS and healthy matched controls.

miRNA	Fold difference
**ME/CFS T0 versus CTRLS T0**	
hsa-miR-29a-3p	+ 2.00*
hsa-miR-150-5p	+ 2.86*
hsa-miR181b-5p	+ 2.77***
hsa-miR-4433a-5p	− 6.46***
hsa-miR-6819-3p	− 11.13***
**ME/CFS T90 versus CTRLS T90**	
hsa-miR-127-3p	+ 2.86*
hsa-miR-140-5p	+ 3.74***
hsa-miR-150-5p	+ 2.93*
hsa-miR-374b-5p	+ 2.49***
hsa-miR-486-5p	− 2.13***
hsa-miR-3620-3p	− 2.47*
hsa-miR-5581-5p	+ 2.18*
hsa-miR-6076	+ 2.39*
hsa-miR-6507-3p	− 3.43*
hsa-miR-6717-5p	+ 3.68*
hsa-miR-6800-3p	− 2.88*
hsa-miR-6875-5p	+ 8.58*
**ME/CFS T90 versus ME/CFS T0**	
hsa-miR-28-5p	+ 2.57***
hsa-miR-29a-3p	+ 1.98*
hsa-miR-140-5p	+ 2.79***
hsa-miR-374b-5p	+ 2.43**
hsa-miR-486-5p	− 2.03***
hsa-miR-6800-3p	− 2.65*
hsa-miR-6875-5p	+ 4.02*
**CTRLS T90 versus CTRLS T0**	
hsa-miR-3620-3p	+ 3.72*
hsa-miR-6507-3p	+ 5.62*

MiRNA expression profile using microarray analysis at baseline and T90 of ME/CFS and healthy matched controls. The data is represented as a fold difference.

ANOVA was used to analyze the differences and measure the significance *P value < 0.05, **P value < 0.01, and ***P value < 0.001.

### Independent validation and replication assays of identified miRNAs

The expression levels of the seventeen circulating miRNAs previously identified with our discovery cohort were validated by quantitative reverse transcription PCR (RT-qPCR) in an independent replication cohort and quantified by fold difference. We successfully replicated 11 out of the 17 identified miRNAs. We were unable to detect the expression levels of six miRNAs (hsa-miR-5581-5p, hsa-miR-6076, hsa-miR-6717-5p, hsa-miR-6875-5p, hsa-miR-6800-3p and hsa-miR-6507-3p) because the qPCR signal was too low to obtain a good, reproducible quantification. For the replicated eleven miRNAs, the expression levels of hsa-miR-28-5p, hsa-miR-127-3p, hsa-miR-140-5p, hsa-miR-374b-5p, hsa-miR4433a-5p and hsa-miR-6819-3p were found to be significantly higher in ME/CFS patients compared to the healthy control group at baseline (*P* < 0.05). The expression levels of hsa-miR-150-5p, hsa-miR-486-5p and hsa-miR-3620-3p were significantly higher in ME/CFS patients compared to healthy controls after 90 min of stimulation (*P* < 0.05) (Table 3). Of note, there were no significant differences observed in the change of expression of these eleven miRNAs between women and men in either the ME/CFS or the control group (Supplementary Table S2).

**Table 3:** miRNA expression differences between ME/CFS group and healthy controls by RT-qPCR in the replication phase.

miRNA	T0	T90
	Fold difference	Fold difference
hsa-miR-28-5p	2.24 ± 0.47*	2.65 ± 0.97
hsa-miR-29a-3p	1.71 ± 0.33	1.73 ± 0.33
hsa-miR-127-3p	4.27 ± 0.78**	2.86 ± 0.47
hsa-miR-140-5p	3.08 ± 0.61**	2.00 ± 0.32
hsa-miR-150-5p	3.37 ± 1.05	8.61 ± 2.53*
hsa-miR-181b-5p	1.56 ± 0.29	1.72 ± 0.40
hsa-miR-374b-5p	2.70 ± 0.56*	2.00 ± 0.35
hsa-miR-486-5p	1.68 ± 0.20	3.02 ± 0.71*
hsa-miR-3620-3p	1.36 ± 0.15	3.30 ± 0.76*
hsa-miR-4433a-5p	1.87 ± 0.29*	2.37 ± 0.77
hsa-miR-6819-3p	2.18 ± 0.32*	2.47 ± 0.38

miRNA expression profile differences between ME/CFS patients and matched controls in the replication phase. Data are shown at baseline (T0)and after stress test (T90)by fold difference.

All data are represented as mean ± standard error of the mean. The results were considered significant at *P value < 0.05, and **P value < 0.01.

### ME/CFS-associated miRNAs and symptom severity

**Figure 2:**
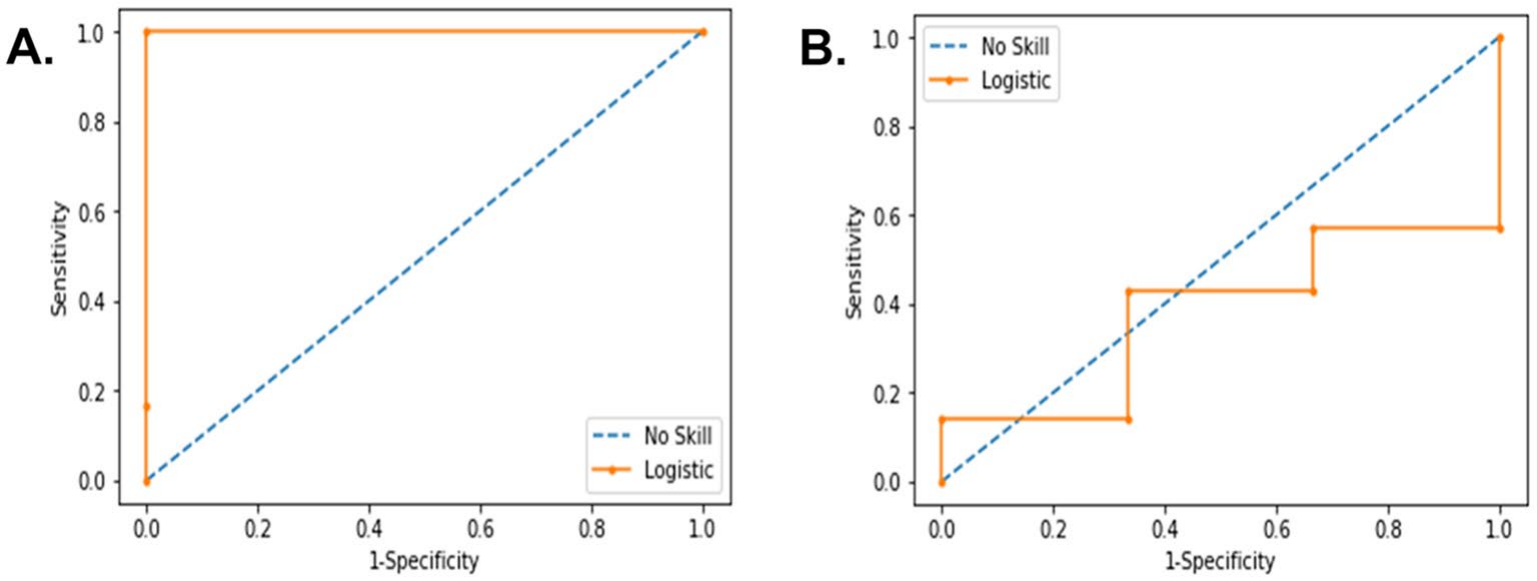
ROC curve analysis for the prediction of ME/CFS using the eleven-miRNA panel (**A**) ROC curve analysis of ΔΔCT. ROC-curve showed perfect predictive capability. ROC AUC (Logistic Curve) = 1. **(B) **ROC curve analysis of ΔCT at baseline. ROC-curve showed unacceptable predictive capability. ROC AUC (Logistic Curve) = 0.381.

**Figure 3:**
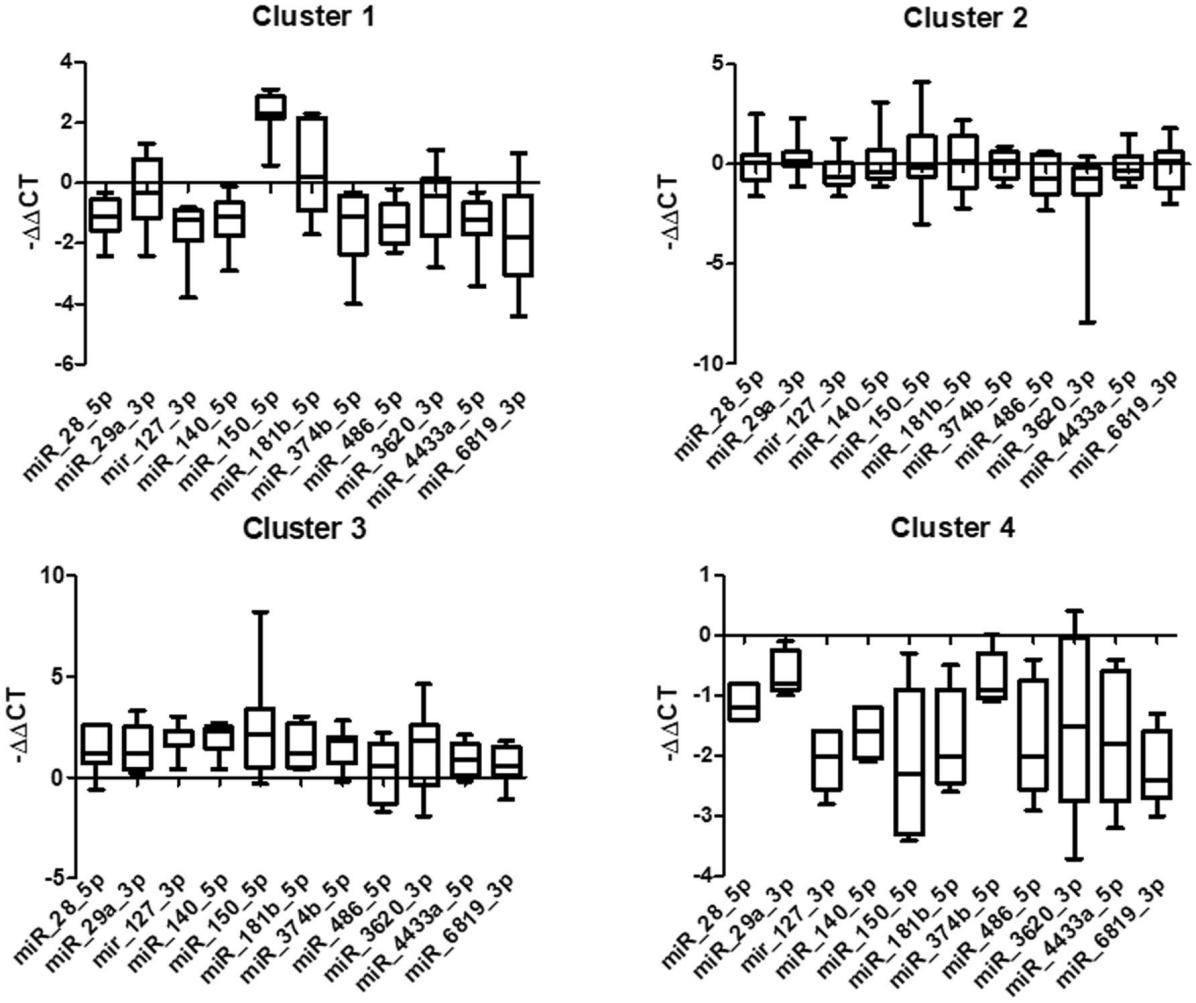
Comparison of the expression of the eleven miRNAs in the four ME/CFS clusters. All eleven miRNAs were upregulated in cluster 3 and downregulated in cluster 4. One-way ANOVA was used to analyze the differences. Results were considered significant at *P*-value < 0.05.

**Figure 4:**
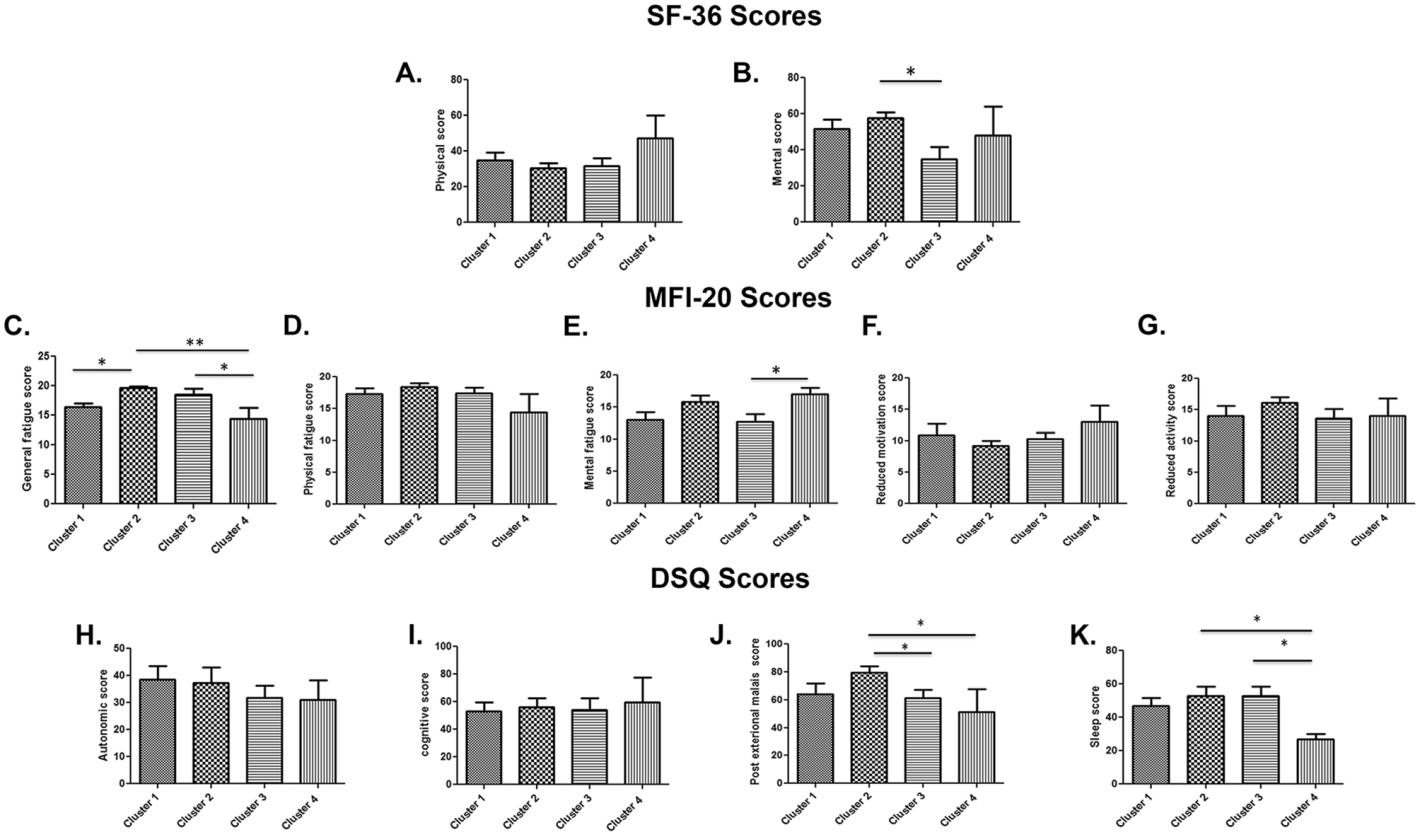
Standard questionnaires among four ME/CFS clusters, including scores for SF-36 (panels A, B); MFI-20 (panels C, D, E, F and G) and DSQ (panels H, I, J and K)). All data are represented as mean ± standard error of the mean. The data were analyzed using one-way ANOVA; and results were considered significant at *P*-value < 0.05 (*).

After the validation step, we evaluated whether these eleven circulating miRNAs were sufficient to diagnose ME/CFS and if their differential expression profiles were associated with typical ME/CFS symptoms and/or symptom severity using the Random Forest Model (RFM). We applied the RFM to RT-qPCR derived ΔCT data (corresponding to the baseline miRNA expression values only) obtained with the training dataset, corresponding to 80% of our patient cohort, and RT-qPCR ΔΔCT data (corresponding to differential miRNA expression values after stimulation versus baseline expression values) using the same training dataset. The results indicated an excellent performance of our RFM with the ΔΔCT using a testing dataset corresponding to 20% of our patient cohort (untested ME/CFS cases). We obtained an accuracy of 90%, a sensitivity of 100%, a specificity of 75% and precision of 86%, with a ROC curve AUC = 1 (Fig. 2A). These results contrasted significantly to those applying the RFM to the ΔCT dataset at baseline alone (Fig. 2B). We then applied the K-means method to the combination of the expression level changes of the eleven miRNAs, which led to an unbiased automated classification of ME/CFS patients into four distinct clusters. A significant difference in the ΔΔCT values for each of the eleven miRNAs between the four clusters was obtained (*P* < 0.05). We observed a distinct miRNA profile in each of the four clusters (Fig. 3). In cluster 1, all the miRNAs were downregulated except for hsa-miR-150-5p and hsa-miR-181b-5p, which were both upregulated. In cluster 2, six miRNAs were upregulated, and five miRNAs were downregulated. In cluster 3, all eleven miRNAs were upregulated, while in cluster 4, they were all downregulated (Fig. 3). Then, we analyzed the differences in ME/CFS score symptoms among the four clusters using the self-reported questionnaires (SF-36, MFI-20, and DSQ) (Fig. 4). We observed that ME/CFS patients classified in cluster 2 and 3 had more severe symptoms when compared to the individuals classified in clusters 1 and 4. In particular, ME/CFS patients classified in cluster 2 exhibited worse general fatigue scores according to the MFI-20 questionnaire (Fig. 4C), presented the most severe PEM and severe sleep disturbances scores (Fig. 4J,K respectively) according to the DSQ questionnaire. Indeed, these housebound patients reported also having a significant decreased daily activity with only 2.9 ± 1.2 h (*P* < 0.05), compared to the ME/CFS patients classified into the other three clusters (Supplementary Table S3). Of note, the ME/CFS patients classified into cluster 2 exhibited a greater number of comorbidities when compared to the other clusters (Supplementary Table S4). Despite that the ME/CFS patients classified into cluster 1 and 4 had milder symptoms, those in cluster 4 exhibited the worse mental fatigue score according to the MFI-20 questionnaire when compared with the other clusters (Fig. 4E). Exploration of the clinical and demographic data among the participants in term of age, BMI, sex, and illness duration did not reveal any significant difference between the four clusters (Supplementary Table S3).

### Gene pathways and networks

**Figure 5:**
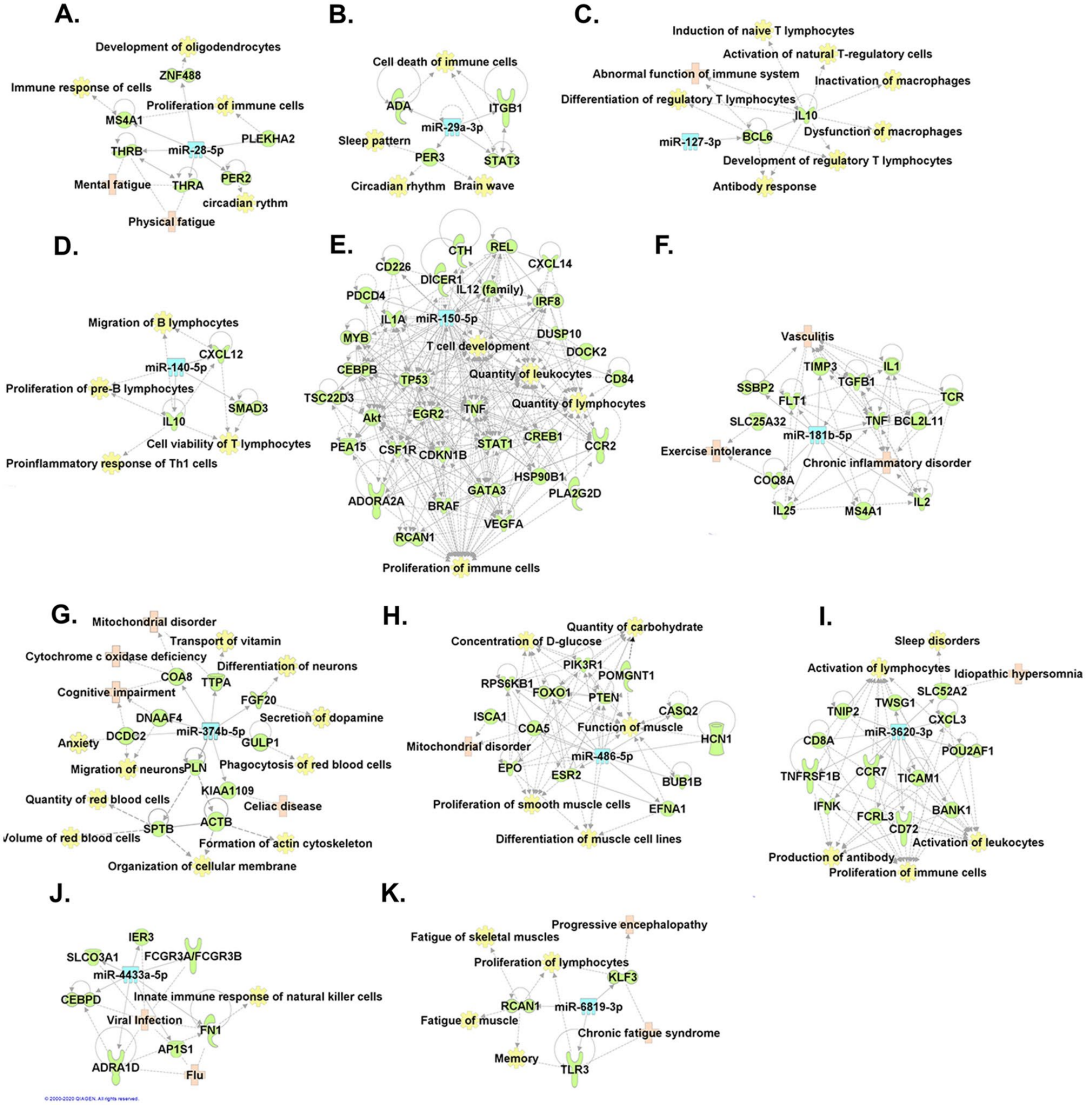
Predicted gene pathway and network of each miRNA. The miRNAs are represented in blue; the genes that are predicted to interact are in green; the diseases that are associated with miRNAs or genes are in light pink; and the molecular and physiological functions are in yellow. The hybrid approach of Ingenuity Pathway Analysis (IPA) software (QIAGEN Inc. software version 51,963,813) and manual curations were applied to construct the networks of hsa-miR-28-5p **(A)**, hsa-miR-29a-3p **(B)**, hsa-miR-127-3p (**C)**, hsa-miR140-5p **(D)**, hsa-miR-150-5p **(E)**, hsa-miR-181b-5p **(F)**, hsa-miR-374b-5p **(G)**, hsa-486-p5 **(H)**, hsa-miR3620-3p **(I)**, hsa-miR-4433a-5p **(J)**, and hsa-miR-6819-3p **(K)**.

**Figure 6:**
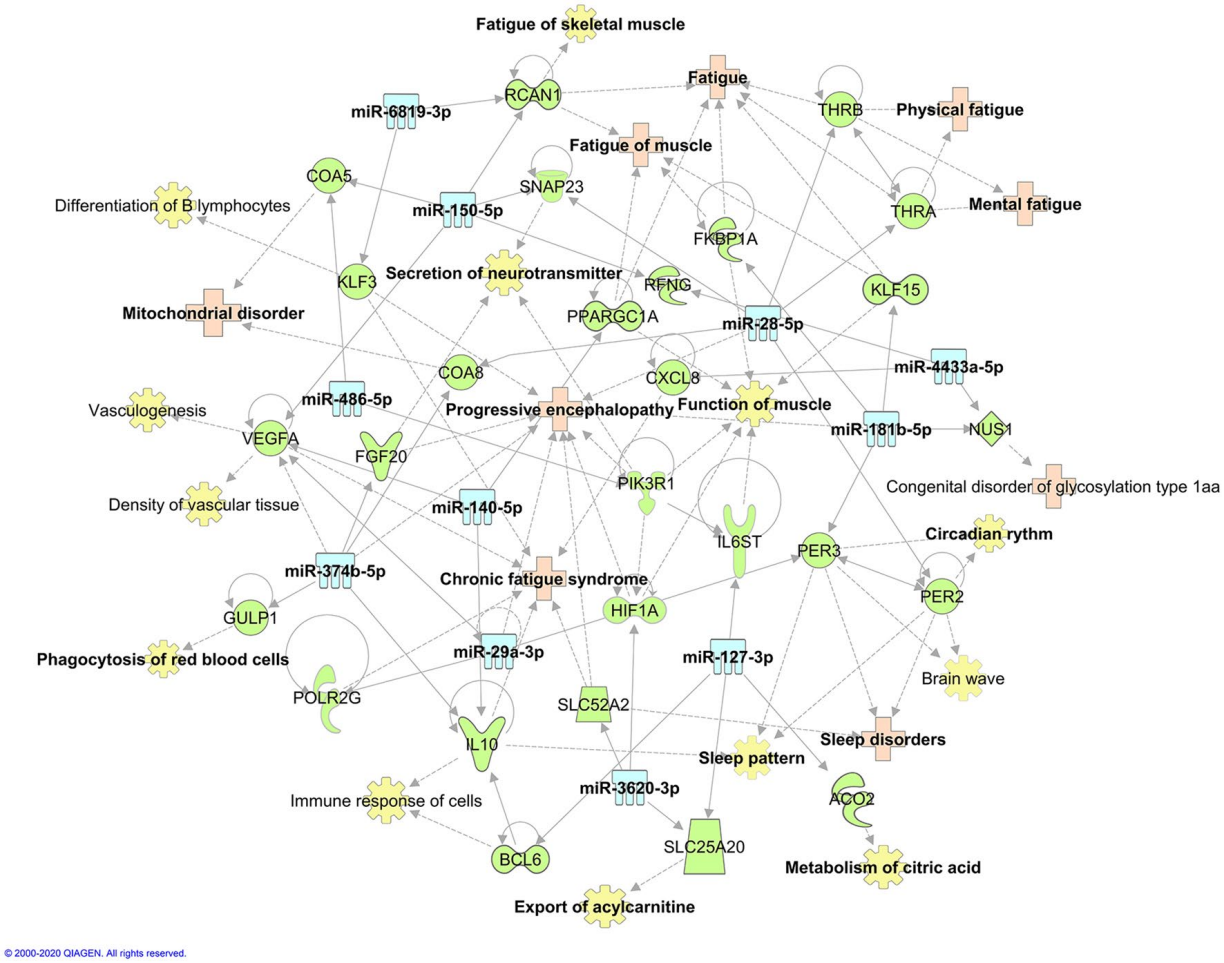
Predicted pathway and network of all the 11 miRNAs. The potential targets of these 11 miRNAs, including genes, molecular and physiological functions, and ME/CFS related diseases or symptoms, are shown in one integrated network. The miRNAs are represented in blue; the genes that are predicted to interact are in green; the diseases that are associated with miRNAs or genes are in light pink; and the molecular and physiological functions are in yellow. The Ingenuity Pathway Analysis (IPA) software (QIAGEN Inc. software version 51,963,813) and manual curations were applied to construct the network.

ME/CFS is a multi-system disease involving the immune system, energy production, and brain. To better understand the mechanistic roles of our identified miRNAs on the molecular functions and physiological symptoms of ME/CFS, we conducted systematic gene pathway and network analyses. As ME/CFS is under-studied for genetics and epigenetics, there is limited knowledge for ME/CFS-related gene networks in existing databases. Nevertheless, our gene pathway analyses using the Ingenuity Pathway Analysis (IPA) software showed that seven of the 11 miRNAs were involved in immune responses or inflammation and one was involved in the muscular system. We then applied a hybrid approach of both IPA and manual curations. We first searched the literature and manually identified genes as well as molecular and physiological functions that have been reported to be associated with ME/CFS, and then built connections with each of the 11 miRNAs based on the IPA experimentally observed Ingenuity Knowledge Base. This comprehensive analysis allowed us to construct more complete networks that connected each miRNA to its targets (e.g., ME/CFS-related genes and physiological functions) that could play critical roles in the pathogenesis of ME/CFS. As expected, our constructed networks revealed multiple key symptoms and features observed in ME/CFS (Fig. 5). Using the IPA and manual curation hybrid approach, we further constructed a larger and more complete network connecting all of these 11 miRNAs and their key genes, functions, and disease targets (Fig. 6).

## Discussion

ME/CFS continues to cause significant morbidity worldwide, and it is estimated that 84–91% of persons with ME/CFS symptoms remain undiagnosed because of the lack of diagnostic biomarkers^19^. Using a two-step strategy, we examined the expression values of miRNAs in the plasma of all enrolled participants (patients with severe ME/CFS and matched healthy controls) at baseline (T0) and after the application of a standardized post-exertional stress challenge (T90). We quantified the changes in miRNA levels between the two time-points using the ∆∆CT method. Our analysis identified eleven miRNAs associated with ME/CFS in response to a post-exertional stress challenge. We established a Random Forest Model using miRNA expression changes (∆∆CT) before and after the post-exertional stress challenge. This unique experimental design allowed the identification of ME/CFS patients versus healthy controls with high accuracy (90%), which ultimately could then be used to predict individuals having ME/CFS. We showed that the biological sex did not influence the miRNAs expression at either baseline or in response to the induction of a PEM. Our results are in sharp contrast with the recent work by Cheema et al.^16^ showing that men and women with ME/CFS exhibit differential miRNA expression profiles in response to exercise. These conflicting findings could be explained primarily by the use of distinct experimental designs, the use of PBMCs vs plasma as well as by the clinical heterogeneity of ME/CFS cases tested (moderate vs severe)^20^. The use of the K-means method allowed us to categorize ME/CFS patients into four clusters according to their miRNA expression profiles and corresponding to changes in the severity of their symptoms (Fig. 4). Indeed, the ME/CFS patients classified in clusters 2 and 3 had the most severe symptoms and a majority of their miRNAs were upregulated. Conversely, the ME/CFS patients classified into clusters 1 and 4 had moderate symptoms, and their miRNAs were downregulated.

The majority of the circulating miRNAs identified in our cohort are involved in the regulation of immunity. Most of them are novel and are for the first time associated with ME/CFS (hsa-miR-28-5p, hsa-miR-29-3p, hsa-miR-181a-5p, hsa-miR-374b-5p, hsa-miR-486-5p, hsa-miR-3620-3p, hsa-miR-4433a-5p, hsa-miR-6819-3p) while few others have been previously reported in other ME/CFS cohorts (hsa-miR-127-3p, hsa-miR-140-5p and hsa-miR-150-5p) and replicated in our study for the first time too. Indeed, hsa-miR-127-3p has been previously reported in an Australian ME/CFS cohort^13^. This miRNA regulates the expression of the *BCL6* gene, which encodes a transcription factor called B-cell lymphoma 6 protein that inhibits the expression of Interleukin 10 (IL-10). This anti-inflammatory cytokine plays a central role in limiting host immune responses to pathogens^21^. It was shown that IL-10 is elevated in the cerebrospinal fluid of some patients suffering from ME/CFS^22^. Previous works from Almenar-Pérez E. et al. have shown an upregulation of hsa-miR-140-5p expression in PBMCs of ME/CFS patients^23^. This miRNA regulates the differentiation of T cells and affects CD4 + T cell metabolism^24^. Moreover, overexpression of hsa-miR-140-5p in some ME/CFS patients could lead to a significant decrease in UL16 protein. This glycoprotein encoded by *ULBP1* gene is responsible for the activation of natural killer (NK) cells and T-lymphocytes via the natural killer group 2, member D membrane receptor NKG2D^25^. More recently, Cheema et al. reporteds the upregulation of hsa-miR-150-5p in PBMCs of ME/CFS patients in response to exercise^16^. This miRNA is known to be associated with the modulation of immunity and inflammatory response^26–28^, while this miRNA is predicted to regulate many genes that participate in the proliferation of immune cells (Fig. 5E).

Among the novel circulating miRNAs differently expressed in the present study, hsa-miR-28-5p and hsa-miR-29a-3p are significantly associated with CD4 + T cell count^29^. Of note, hsa-miR-28-5p is predicted to target *MS4A1* (CD20) and *PLEKHA2* (Pleckstrin homology domain-containing family A member 2) genes, which are known to participate in the immune cell responses and stimulate their differentiation^30,31^. Similarly, hsa-miR-29a-3p is predicted to target genes such as *ADA* (Adenosine Deaminase), *ITGB1* (Integrin Subunit Beta 1) and *STAT3* (Signal transducer and activator of transcription 3), which are involved in many cellular functions including the death of immune cells^32–34^. It should be noted that overexpression of hsa-miR-29a-3p in ME/CFS patients may contribute to the reduction of their ability to respond to certain viral infections by targeting RNase L (ribonuclease L), which is known among others, for its central role in innate immunity. Moreover, RNase L plays a vital role in the modulation of antiviral and anti-proliferative activities mediated by interferon^26,35^. Furthermore, the expression of hsa-miR-181b-5p is reduced in invariant NK T cell-deficient mice^36^ and interestingly, severely ill ME/CFS patients also exhibited alteration in their invariant NK T cells^37,38^. Altered expression of hsa-miR-4433a-5p was previously reported in serum samples of influenza H7N9 infected patients^39^. Since many ME/CFS patients reported that their disease onset followed a viral infection, this miRNA is of interest given that its predicted targets are involved in the regulation of viral responses (Fig. 5J). Hsa-miR-6819-3p is another miRNA predicted to participate in the abnormal immune responses and PEM occurring in ME/CFS. It is worth mentioning that this miRNA also targets genes that have previously been related to ME/CFS, *KLF3* (Krüppel-like factor 3) and *TLR3* (Toll-like receptor 3)^40,41^ (Fig. 5K). Rintatolimod, also known commercially as Ampligen, is a dsRNA that functions as an activating ligand for *TLR3* and has been tested in many clinical trials as a treatment for ME/CFS^40^. Therefore, high expression of hsa-miR-6819-3p could reduce the efficacy of Rintatolimod and might explain the non-responsiveness toward this drug, as previously observed in some ME/CFS patients.

Circulating miRNAs are also altered by exercise and could represent useful biomarkers to characterize PEM occurring in ME/CFS and therapeutic targets to prevent or manage this condition. Among the miRNAs associated with the physiological responses to exercise and post-exertion, Makarova et al. suggested that hsa-miR-181b-5p might play several roles in adapting to physical efforts as an endurance regulator^42,43^. Shah et al. have reported an overexpression of hsa-miR-181b-5p in participants’ plasma after exercise^44^. Indeed, exposure of mice to acute exercises resulted in a significant increase in mmu-miR-181b-5p expression in skeletal muscle tissue in young (but not older) mice. Previous work revealed a strong role for hsa-miR-181b-5p in vascular inflammation in obesity, insulin resistance, sepsis, and cardiovascular disease^44^. Therefore, these results suggest that hsa-miR-181b-5p may play a role in dampening inflammation in response to acute exercise (Fig. 5F). Similarly, expression of hsa-miR-486-5p in response to regular exercise resulted in significantly increased expression in sedentary old men^45^, contrasting with healthy adults^46^ showing rather a decrease in hsa-miR-486-5p expression. Of note, acute exercice generates a significant increase in hsa-miR-486-5p expression in young men^47^. Interestingly, upregulation of hsa-miR-486-5p expression in endurance athletes positively correlates with VO_2_ max values^48^. Among other miRNAs associated response to exercise, hsa-miR-3620-3p is the most overexpressed circulating miRNA in endurance athletes^49^, and its expression is increased in ME/CFS patients, whereas it is decreased in control subjects after the application of our post-exertional stress challenge. This miRNA targets genes involved in the regulation of circadian clock (Fig. 5I) like *PER* 2 (period circadian protein homolog 2) and *PER3* (period circadian protein homolog 3), and could be involved in sleep disturbances occurring in ME/CFS patients^50,51^.

In the present study, we showed higher expression of hsa-miR-374b-5p in the plasma of ME/CFS compared to healthy controls at baseline, which contradicts the findings of a previous study showing the opposite but which could be explained by the use of PBMCs instead of plasma samples^16^. Hsa-miR-374b-5p targets many genes predicted to be involved in many ME/CFS symptoms, including mitochondrial dysfunctions, wheat sensitivity, fatigue and vitamin E metabolism^36–38,44^ (Fig. 5G). Moreover, high hsa-miR-374b-5p expression levels at baseline or after PEM induction could play an essential role in the regulation of red blood cells (RBCs) shape and membrane deformability by targeting the *SPTB* and *ACTB* genes, which encode β-spectrin and β-actin proteins, respectively^52^. This is of interest given that RBCs of ME/CFS patients are significantly larger and less deformable compared to those of healthy individuals^53^.

Among possible limitations, longitudinal studies must be undertaken to characterize the variability of PEM development, symptom severity and duration following the application of our post-exertional stress challenge. While severely affected persons with ME/CFS (housebound or bedridden) cannot be tested by CPET approach, it would be interesting to compare mild to moderately affected patients using both methods to establish their sensitivity and limitations using our panel of circulating microRNAs.

In conclusion, we developed a post-exertional stress challenge that provokes PEM in ME/CFS patients. Measurement of the differential expression of circulating miRNAs in severely affected ME/CFS patients led to the discovery and validation of eleven miRNAs associated with ME/CFS. Based on these different miRNA signatures, machine learning algorithm led to the classification of ME/CFS patients into four clusters associated with symptom severity. These findings may provide a foundation for the development of a new non-invasive test to diagnose ME/CFS patients. These miRNA signatures and clusters could eventually be used to predict responses to pharmacological treatments for ME/CFS, and may even allow clinicians to identify individuals to whom such treatments could be beneficial. In addition, we present possible mechanisms that still need to be validated, by which each of the miRNAs could play a role in the pathogenesis and etiology of ME/CFS.

## Materials and methods

### Study populations

Forty-three patients with ME/CFS and twenty-five age- and sex-matched healthy controls were recruited for this study (Table 1). The ME/CFS patients were diagnosed using the Canadian consensus criteria*.* The healthy control subjects had no family history or symptoms of ME/CFS. The protocol of this study was approved by the Institutional Review Board of Sainte-Justine University Hospital (protocol #4047). Written informed consent was obtained from each participant. All experiments were performed following relevant guidelines and human ethic regulations.

### Evaluation of ME/CFS symptoms and participant health status

All participants completed standard questionnaires, including 36-Item Short-Form Health Survey (SF-36), Multidimensional Fatigue Inventory (MFI-20), and the DePaul Symptom Questionnaire (DSQ)^54–56^, to assess their health status and symptoms of ME/CFS^56^. The SF-36 scaled scores provide a physical health score and a mental health score, while the MFI-20 scores are stratified into General Fatigue, Physical Fatigue, Reduced Activity, Reduced Motivation, and Mental Fatigue. The DSQ, which provides 54 summed values to assess the health status, were grouped into four factors: Neuroendocrine, Autonomic and Immune Dysfunction; Cognitive Dysfunction; Post-exertional Malaise; and Sleep Disturbances^56^.

### Post-exertional stress challenge

The participants were exposed to a stress-test to provoke PEM using an ABR therapeutic massager device developed by Panacis Medical Ltd. (Ottawa, Ontario, Canada). The ABR method includes an inflatable cuff that is applied to the arm of each participant. The cuff dynamically exerts pulsatile compressions producing a pressure of variable amplitude from 0–4 psi at 0.006 Hz. All participants were mechanically stimulated for 90 min (T90) to induce PEM and evaluate changes in the miRNA expression profile in response to this mechanical stimulation.

### Blood specimen collection

Peripheral blood samples of participants were collected at two time-points (baseline, T0 and after the stress-test, T90) in EDTA-treated tubes, and centrifuged at 11,000×*g* for 10 min. Derived plasma samples were aliquoted and kept frozen at − 80 °C until analysis.

### RNA extraction for microRNA array analysis

MiRNAs were extracted from plasma samples obtained from ME/CFS patients (n = 11), and matched healthy controls (n = 8) as illustrated in Fig. 1A. Plasma samples were thawed and centrifugated at 17,000×*g* for 15 min at 4 °C. The RNA was extracted using the miRNEASY kit (miRNeasy Serum/Plasma Kit, Qiagen, Hilden, Germany) according to the manufacturer's instructions.

### Microarray analysis

A global expression profiling was performed for each participant in the discovery cohort at Genome Quebec Innovation Center (Montreal, QC, Canada), using the Agilent expression array-Human miRNA 8 × 60 K (Agilent Technologies, Santa Clara, CA, USA) harboring 2549 human miRNAs. We selected only miRNAs exhibiting ± two-fold changes with a false discovery rate (FDR) < 0.005 using the SpringGene software by Agilent, in combination with quantile normalization.

### Validation of candidate miRNAs in replication cohort by qPCR

The plasma samples from the replication cohort were thawed on ice for 15 min, followed by centrifugation for 15 min at 17,000×*g* at 4 °C to remove any remaining cellular debris. RNA extraction with enrichment of small RNAs was performed using the mirVana PARIS extraction kit (mirVanaPARIS RNA and Native Protein Purification Kit, Thermo Fisher Scientific, Waltham, MA, USA) according to manufacturer's instructions. 75 µl of eluent solution was used to elute the RNAs from the filter cartridge, and RNA samples were stored at − 80 °C. As a spike-in control, 50 nmol of Cel-miR-39-3p synthetic oligonucleotide RNA with the sequence: UCACCGGGUGUAAAUCAGCUUG (Thermo Fisher Scientific) was added to the plasma after addition of denaturing solution.

### Complementary DNA (cDNA) synthesis, qPCR miRNA detection and quantification

cDNA was synthesized from the extracted miRNA samples using a PCR thermocycler (T3000 Thermocycler, Biometra, Montreal Biotech Inc, Montreal, QC, Canada) and the TaqMan Advanced miRNA cDNA Synthesis Kit (Thermo Fisher Scientific) by following manufacturer's instructions. The resulting cDNA samples were stored at − 20 °C. The synthesized cDNA was the template for qPCR using the TaqMan Advanced miRNA Assays (Thermo Fisher Scientific) and probes for each miRNA. The qPCR reaction was performed using the QuantStudio 3 instrument (Thermo Fisher Scientific). The qPCR was performed in duplicate for each sample, and the mean of the obtained cycle thresholds (CT) was used for calculations.

### qPCR data analysis

The expression levels of miRNAs in response to the stress challenge were comparatively quantified using the ΔΔCT method. The ΔCT was first calculated by subtracting the CT value of each miRNA from that of the internal control, cel-mir-39a-3p (ΔCT at T0 = CTmiR _T0_ − CTcel-miR-39a-3p _T0_). The results of miRNAs at T90 were normalized in the same way, where ΔCT_T90_ = CTmiR _T90_ − CTcel-miR-39a-3p _T90_. Finally, miRNA expression levels in response to the test of the same patient were evaluated by calculating the ΔΔCT as follows: ΔΔCT = ΔCT_T90_ − ΔC_T0_. The fold difference between the expression of each miRNA in each sample to the mean expression of the controls was analyzed using the 2^- ΔΔCT^ method at two timepoints, T0 and T90. First, the results of each miRNA for each sample were normalized with the results of the exogenous control, cel-mir-39a-3p, where ΔCT_sample_ = CT_miR_ − CT_cel-miR-39a-3p_. Then, the ΔΔCT was calculated for each sample as follows, ΔΔCT = ΔCT_sample_ − mean ΔCT_CTRLs_. Finally, the fold difference in miRNA expression between each participant and the mean of controls was calculated as 2^−ΔΔCT^.

### Construction of gene pathways and networks targeted by dysregulated miRNAs in ME/CFS

The potential targets of miRNAs of interest, including genes, molecular and physiological functions, and ME/CFS-related diseases and symptoms, were primarily identified through comprehensive literature reviews and manual curations. The connections (interactions) of the miRNAs and their targets were constructed based on the Ingenuity Knowledge Base using the Ingenuity Pathway Analysis (IPA) software (QIAGEN Inc. software version 51963813).

### Machine learning and statistical analyses

Random Forest Model (RFM) was performed to predict individuals affected with ME/CFS. The data was randomly split 80/20 into training and testing sets. RFM model was built using the training data and subsequently tested on the remaining 20% of data making up the testing set. To evaluate the RFM, we used different measures to assess the classification performance, including accuracy, specificity, sensitivity, and receiver operating characteristic (ROC) curve. The ROC curve showed the trade-off between sensitivity and specificity, and the area under the curve (AUC) was used as an index for evaluating the predictive performance of the constructed eleven miRNA panel. We applied the K-means method to classify the subjects into four clusters using the ΔΔCT data based on the signature of the eleven miRNAs. The goal of K-means is to define clusters of patients, so we can derive insights about their symptoms and other clinical characteristics. ME/CFS symptom data from questionnaires regarding these four clusters were analyzed using ANOVA, followed by Tukey multiple comparison tests. P values less than 0.05 were considered to be statistically significant.

## Supplementary information

Supplementary Information.

## Data Availability

The datasets generated and analysed during the current study are available from the corresponding author on reasonable request.

## References

[1] Missailidis D, Annesley SJ, Fisher PR (2019). Pathological mechanisms underlying myalgic encephalomyelitis/chronic fatigue syndrome. Diagnostics.

[2] Carruthers BM (2003). Myalgic encephalomyelitis/chronic fatigue syndrome: clinical working case definition, diagnostic and treatment protocols. J.Chronic Fatigue Syndrome.

[3] Cortes Rivera M, Mastronardi C, Silva-Aldana CT, Arcos-Burgos M, Lidbury BA (2019). Myalgic encephalomyelitis/chronic fatigue syndrome: a comprehensive review. Diagnostics.

[4] Capelli E (2010). Chronic Fatigue Syndrome/Myalgic Encephalomyelitis: An Update.

[5] Anglicheau D, Muthukumar T, Suthanthiran M (2010). MicroRNAs: small RNAs with big effects. Transplantation.

[6] Greene J (2017). Circular RNAs: biogenesis, function and role in human diseases. Front. Mol. Biosci..

[7] MacFarlane L-A, Murphy R (2010). MicroRNA: biogenesis, function and role in cancer. Current Genom..

[8] 8Bolha, L., Ravnik-Glavač, M. & Glavač, D. Circular RNAs: biogenesis, function, and a role as possible cancer biomarkers. *Int. J. Genom. *(2017). 10.1155/2017/6218353 PMC5733622 29349062

[9] Maciotta Rolandin S, Meregalli M, Torrente Y (2013). The involvement of microRNAs in neurodegenerative diseases. Front. Cell. Neurosci..

[10] 10Quinlan, S., Kenny, A., Medina, M., Engel, T. & Jimenez-Mateos, E. M. in *International review of cell and molecular biology*. **334**, 309–343 ( 2017). 10.1016/bs.ircmb.2017.04.002 28838542

[11] 11Sharma, S. & Lu, H.-C. microRNAs in neurodegeneration: current findings and potential impacts. *Journal of Alzheimer's disease & Parkinsonism* **8** (2018). 10.4172/2161-0460.1000420 PMC5976447 29862137

[12] Condrat CE (2020). miRNAs as biomarkers in disease: latest findings regarding their role in diagnosis and prognosis. Cells.

[13] Brenu EW, Ashton KJ, Batovska J, Staines DR, Marshall-Gradisnik SM (2014). High- throughput sequencing of plasma microRNA in chronic fatigue syndrome/myalgic encephalomyelitis. PLoS ONE.

[14] Brenu EW (2012). Cytotoxic lymphocyte microRNAs as prospective biomarkers for chronic fatigue syndrome/myalgic encephalomyelitis. J. Affect. Disord..

[15] Petty RD, McCarthy NE, Le Dieu R, Kerr JR (2016). MicroRNAs hsa-miR-99b, hsa-miR-330, hsa-miR-126 and hsa-miR-30c: potential diagnostic biomarkers in natural killer (NK) cells of patients with chronic fatigue syndrome (CFS)/myalgic encephalomyelitis (ME). PLoS ONE.

[16] Cheema AK (2020). Unravelling myalgic encephalomyelitis/chronic fatigue syndrome (ME/CFS): Gender-specific changes in the microRNA expression profiling in ME/CFS. J. Cell Mol. Med..

[17] Takakura S, Oka T, Sudo N (2019). Changes in circulating microRNA after recumbent isometric yoga practice by patients with myalgic encephalomyelitis/chronic fatigue syndrome: an explorative pilot study. BioPsychoSocial Med..

[18] Baraniuk JN, Shivapurkar N (2017). Exercise–induced changes in cerebrospinal fluid miRNAs in Gulf War Illness, Chronic Fatigue Syndrome and sedentary control subjects. Sci. Rep..

[19] Jason LA, Zinn LM, Zinn AM (2015). Myalgic encephalomyelitis: symptoms and biomarkers. Curr. Neuropharmacol..

[20] Valdez AR (2019). Estimating prevalence, demographics, and costs of ME/CFS using large scale medical claims data and machine learning. Front. Pediatrics.

[21] Saito Y (2006). Specific activation of microRNA-127 with downregulation of the proto-oncogene BCL6 by chromatin-modifying drugs in human cancer cells. Cancer Cell.

[22] Natelson BH, Weaver SA, Tseng C-L, Ottenweller JE (2005). Spinal fluid abnormalities in patients with chronic fatigue syndrome. Clin. Diagn. Lab. Immunol..

[23] Almenar-Pérez E, Sarría L, Nathanson L, Oltra E (2020). Assessing diagnostic value of microRNAs from peripheral blood mononuclear cells and extracellular vesicles in Myalgic Encephalomyelitis/Chronic Fatigue Syndrome. Sci. Rep..

[24] Zhu S (2019). miR-140-5p regulates T cell differentiation and attenuates experimental autoimmune encephalomyelitis by affecting CD4+ T cell metabolism and DNA methylation. Int. Immunopharmacol..

[25] Himmelreich H, Mathys A, Wodnar-Filipowicz A, Kalberer CP (2011). Post-transcriptional regulation of ULBP1 ligand for the activating immunoreceptor NKG2D involves 3′ untranslated region. Hum. Immunol..

[26] Ying W (2016). miR-150 regulates obesity-associated insulin resistance by controlling B cell functions. Sci.Rep..

[27] Kroesen BJ (2015). Immuno-miRs: critical regulators of T-cell development, function and ageing. Immunology.

[28] Zhou B, Wang S, Mayr C, Bartel DP, Lodish HF (2007). miR-150, a microRNA expressed in mature B and T cells, blocks early B cell development when expressed prematurely. Proc. Natl. Acad. Sci..

[29] Qi Y (2017). MicroRNA profiling in plasma of HIV-1 infected patients: potential markers of infection and immune status. J Public Health Emerg.

[30] Engelberts PJ (2016). Type I CD20 antibodies recruit the B cell receptor for complement-dependent lysis of malignant B cells. J. Immunol..

[31] Landego I (2012). Interaction of TAPP adapter proteins with phosphatidylinositol (3, 4)-bisphosphate regulates B-cell activation and autoantibody production. Eur. J. Immunol..

[32] Van De Wiele CJ (2006). Further differentiation of murine double-positive thymocytes is inhibited in adenosine deaminase-deficient murine fetal thymic organ culture. J. Immunol..

[33] Ding C (2016). STAT3 signaling in B cells is critical for germinal center maintenance and contributes to the pathogenesis of murine models of lupus. J. Immunol..

[34] Saito Y (2010). Apoptotic death of hematopoietic tumor cells through potentiated and sustained adhesion to fibronectin via VLA-4. J. Biol. Chem..

[35] Bisbal C, Silverman RH (2007). Diverse functions of RNase L and implications in pathology. Biochimie.

[36] de Candia P (2016). The circulating microRNome demonstrates distinct lymphocyte subset-dependent signatures. Eur. J. Immunol..

[37] Hardcastle SL (2015). Longitudinal analysis of immune abnormalities in varying severities of chronic fatigue syndrome/myalgic encephalomyelitis patients. J. Transl. Med..

[38] Ramos S (2016). Regulatory T, natural killer T and gammadelta T cells in multiple sclerosis and chronic fatigue syndrome/myalgic encephalomyelitis: a comparison. Asian Pac J Allergy Immunol.

[39] Peng F, Loo JFC, Kong SK, Li B, Gu D (2017). Identification of serum MicroRNAs as diagnostic biomarkers for influenza H7N9 infection. Virol. Rep..

[40] Mitchell WM (2016). Efficacy of rintatolimod in the treatment of chronic fatigue syndrome/myalgic encephalomyelitis (CFS/ME). Exp. Rev. Clin. Pharmacol..

[41] Karczewski KJ (2013). Systematic functional regulatory assessment of disease-associated variants. Proc. Natl. Acad. Sci..

[42] Radom-Aizik S (2012). Effects of exercise on microRNA expression in young males peripheral blood mononuclear cells. Clin. Transl. Sci..

[43] Makarova JA (2014). Exercise immunology meets MiRNAs. Exerc. Immunol. Rev..

[44] Shah R (2017). Small RNA-seq during acute maximal exercise reveal RNAs involved in vascular inflammation and cardiometabolic health: brief report. Am. J. Physiol. Heart Circul. Physiol..

[45] Nair VD (2020). Sedentary and trained older men have distinct circulating exosomal microRNA profiles at baseline and in response to acute exercise. Front. Physiol..

[46] Barber JL (2019). The effects of regular exercise on circulating cardiovascular-related microRNAs. Sci. Rep..

[47] D’Souza RF (2018). Circulatory exosomal miRNA following intense exercise is unrelated to muscle and plasma miRNA abundances. Am. J. Physiol. Endocrinol. Metab..

[48] Denham J, Prestes PR (2016). Muscle-enriched microRNAs isolated from whole blood are regulated by exercise and are potential biomarkers of cardiorespiratory fitness. Front. Genet..

[49] Hecksteden A (2016). miRNAs and sports: tracking training status and potentially confounding diagnoses. J. Transl. Med..

[50] Zhang L (2016). A PERIOD3 variant causes a circadian phenotype and is associated with a seasonal mood trait. Proc. Natl. Acad. Sci..

[51] Jackson ML, Bruck D (2012). Sleep abnormalities in chronic fatigue syndrome/myalgic encephalomyelitis: a review. J. Clin. Sleep Med..

[52] Lux SE (2016). Anatomy of the red cell membrane skeleton: unanswered questions. Blood.

[53] Saha AK (2019). Red blood cell deformability is diminished in patients with Chronic Fatigue Syndrome. Clin. Hemorheol. Microcirc..

[54] Framework IC (1992). The MOS 36-item short-form health survey (SF-36). Med. Care.

[55] Smets E, Garssen B, Bonke B, d. & De Haes, J. (1995). The Multidimensional Fatigue Inventory (MFI) psychometric qualities of an instrument to assess fatigue. J. Psychos. Res..

[56] Jason, L. A. *et al.* Factor analysis of the DePaul Symptom Questionnaire: Identifying core domains. *Journal of neurology and neurobiology* **1** (2015). 10.16966/2379-7150.114 PMC4830389 27088131

