# Supplementary material for: Profile of circulating microRNAs in myalgic encephalomyelitis and their relation to symptom severity, and disease pathophysiology
Source: Sci Rep. 2020 Nov 12;10:19620. doi: 10.1038/s41598-020-76438-y (PMC7665057; doi:10.1038/s41598-020-76438-y)
Supplement: Supplementary file 1 — Supplementary Information. [file 41598_2020_76438_MOESM1_ESM.docx]

**Profile of Circulating MicroRNAs in Myalgic Encephalomyelitis and their Relation to Symptom Severity, and Disease Pathophysiology**

Evguenia Nepotchatykh,^a,b,1^ Wesam Elremaly,^a,c,1^ Iurie Caraus,^a,c,1^ Christian Godbout,^d^ Corinne Leveau,^a,c^ Lynda Chalder,^a,c^ Catherine Beaudin,^a^ Emi Kanamaru,^a^ Renata Kosovskaia,^a^ Shawn Lauzon,^a^ Yanick Maillet,^a^ Anita Franco,^a^ Viorica Lascau-Coman,^a^ Saadallah Bouhanik,^a^ Yaned Patricia Gaitan,^a^ Dawei Li,^e^ and Alain Moreau ^a,b,c,f,*^

**Supplementary Information**

**Supplementary Table S1. Post-exertional malaise symptoms reported by ME/CFS patients following the application of the post-exertional stress challenge**

|  | **PEM Symptoms Distribution** |
| --- | --- |
| Profound Fatigue | 23/32 (0.72) |
| Nausea | 3/32 (0.09) |
| Flu symptoms | 4/32 (0.13) |
| Mental fog | 1/32 (0.03) |
| Difficulty concentrating | 2/32 (0.06) |
| Disorientation | 2/32 (0.06) |
| Muscle pain | 5/32 (0.16) |
| Joint pain | 2/32 (0.06) |
| Dizziness | 2/32 (0.06) |
| Headache | 5/32 (0.16) |
| Sleep problems | 4/32 (0.13) |
| Cannot recall | 5/32 (0.16) |
| Other Symptoms | 4/32 (0.13) |

Post-exertional malaise symptom exacerbation reported by ME/CFS patients following the application of our post-exertional stress challenge. The symptoms reported with the greatest frequency by ME/CFS patients following the stress-test were, fatigue (72% of patients), headache (16% of patients) and muscle pain (16% of patients).

**Supplementary Table S2. Comparison of expression level changes of eleven miRNAs in Men and Women in the replication cohort**

| **miRNA** | **ME/CFS Women (N = 18)** | **ME/CFS Men (N = 14)** | **CTRLs Women (N = 11)** | **CTRLs Men (N = 6)** |
| --- | --- | --- | --- | --- |
| hsa-miR-28-5p | -0.01 ± 0.35 | -0.42 ± 0.32 | 0.06 ± 0.22 | -0.30 ± 0.37 |
| hsa-miR-29a-3p | 0.19 ± 0.31 | 0.44 ± 0.30 | 0.66 ± 0.23 | -0.07 ± 0.45 |
| hsa-miR-127-3p | -0.35 ± 0.36 | -0.66 ± 0.46 | -0.33 ± 0.29 | -0.36 ± 0.45 |
| hsa-miR-140-5p | -0.04 ± 0.38 | -0.34 ± 0.42 | 0.21 ± 0.20 | 0.00 ± 0.48 |
| hsa-miR-150-5p | 1.32 ± 0.61 | 0.49 ± 0.62 | 0.50 ± 0.55 | -0.47 ± 0.69 |
| hsa-miR-181b-5p | 0.57 ± 0.36 | -0.04 ± 0.48 | 0.17 ± 0.30 | -0.21 ± 0.51 |
| hsa-miR-374b-5p | -0.18 ± 0.34 | -0.17 ± 0.37 | 0.00 ± 021 | -0.16 ± 0.42 |
| hsa-miR-486-5p | -0.49 ± 0.31 | -1.17 ± 0.26 | -1.34 ± 0.36 | -1.61 ± 0.46 |
| hsa-miR-3620-3p | -0.91 ± 0.53 | -0.08 ± 0.52 | -1.40 ± 0.57 | -0.97 ± 0.58 |
| hsa-miR-4433a-5p | -0.37 ± 0.32 | -0.59 ± 0.32 | -0.52 ± 0.25 | -0.65 ± 0.40 |
| hsa-miR-6819-3p | -0.82 ± 0.42 | -0.93 ± 0.41 | -1.17 ± 0.39 | -0.76 ± 0.60 |

miRNA change of expression differences between ME/CFS men versus ME/CFS women and CTRLs men and CTRLs women. Data are shown by ∆∆CT. All data are represented as mean ± standard error of the mean. The results were considered significant at *P*-value ˂ 0.05 (*). No statistical difference between men and women is observed.

**Supplementary Table S3.** **Clinical and demographic characteristics of the four clusters of ME/CFS patients**

|  | Cluster 1 | Cluster 2 | Cluster 3 | Cluster 4 |
| --- | --- | --- | --- | --- |
| Age (years) | 48.7 ± 3.8 | 53.7 ± 2.3 | 46.9 ± 3.6 | 44 ± 8.9 |
| BMI (kg/m^2^) | 25 ± 1.5 | 24.5 ± 1.2 | 25 ± 1.4 | 26.9 ± 2.6 |
| Male/female | 3/6 | 3/8 | 4/3 | 4/1 |
| Illness duration (years) | 12.3 ± 3.8 | 13.9 ± 3.1 | 13.7 ± 5.6 | 22 ± 6.9 |
| Daily activities(hours) | 4.5 ± 1.7 | 2.9 ± 0.7 * | 6.6 ± 1.5 | 7.9 ± 1.4 |
| Viral /infection | Yes | Yes | Yes | Yes |
| Comorbidities distribution | | | | |
| Allergy | 0/9 (0) | 0/11 (0) | 1/7 (0.14) | 0/5 (0) |
| Anemia | 1/9 (0.11) | 0/11 (0) | 0/7 (0) | 0/5 (0) |
| Anxiety | 2/9 (0.22) | 0/11 (0) | 0/7 (0) | 0/5 (0) |
| Arthritis | 0/9 (0) | 2/11 (0.18) | 0/7 (0) | 0/5 (0) |
| Asthma | 1/9 (0.11) | 1/11 (0.09) | 1/7 (0.14) | 2/5 (0.40) |
| Bronchitis | 0/9 (0) | 1/11 (0.09) | 0/7 (0) | 0/5 (0) |
| Celiac disease | 0/9 (0) | 1/11 (0.09) | 0/7 (0) | 0/5 (0) |
| Cervical subluxation | 0/9 (0) | 1/11 (0.09) | 0/7 (0) | 0/5 (0) |
| Chronic sinusitis | 0/9 (0) | 1/11 (0.09) | 0/7 (0) | 1/5 (0.20) |
| Cystitis | 0/9 (0) | 1/11 (0.09) | 0/7 (0) | 0/5 (0) |
| Cytomegalovirus | 0/9 (0) | 1/11 (0.09) | 0/7 (0) | 0/5 (0) |
| Depression | 0/9 (0) | 0/11 (0) | 0/7 (0) | 2/5 (0.40) |
| Esophagitis | 0/9 (0) | 0/11 (0) | 0/7 (0) | 1/5 (0.20) |
| Glaucoma | 0/9 (0) | 0/11 (0) | 0/7 (0) | 1/5 (0.20) |
| Heart problem | 1/9 (0.11) | 0/11 (0) | 0/7 (0) | 0/5 (0) |
| Heavy metal contamination | 0/9 (0) | 1/11 (0.09) | 0/7 (0) | 0/5 (0) |
| Hip bursitis | 0/9 (0) | 0/11 (0) | 0/7 (0) | 1/5 (0.20) |
| Hypercholesterolemia | 0/9 (0) | 2/11 (0.18) | 0/7 (0) | 0/5 (0) |
| Hypertension | 2/9 (0.22) | 0/11 (0) | 1/7 (0.14) | 0/5 (0) |
| Hyperthyroidism | 0/9 (0) | 0/11 (0) | 0/7 (0) | 1/5 (0.20) |
| Hypoglycemia | 0/9 (0) | 0/11 (0) | 0/7 (0) | 1/5 (0.20) |
| Hypotension | 0/9 (0) | 1/11 (0.09) | 0/7 (0) | 0/5 (0) |
| Hypothyroidism | 0/9 (0) | 1/11 (0.09) | 1/7 (0.14) | 0/5 (0) |
| Intestinal parasites | 1/9 (0.11) | 0/11 (0) | 0/7 (0) | 0/5 (0) |
| Irritable bowel syndrome | 2/9 (0.22) | 0/11 (0) | 1/7 (0.14) | 0/5 (0) |
| Labyrinthitis | 1/9 (0.11) | 0/11 (0) | 0/7 (0) | 0/5 (0) |
| Mastoiditis | 0/9 (0) | 1/11 (0.09) | 0/7 (0) | 0/5 (0) |
| Migraine | 0/9 (0) | 1/11 (0.09) | 0/7 (0) | 1/5 (0.20) |
| Neutropenia | 0/9 (0) | 0/11 (0) | 0/7 (0) | 1/5 (0.20) |
| Ophthalmic irritation | 1/9 (0.11) | 0/11 (0) | 0/7 (0) | 0/5 (0) |
| Pneumonia | 0/9 (0) | 1/11 (0.09) | 0/7 (0) | 0/5 (0) |
| Ranchitism | 1/9 (0.11) | 0/11 (0) | 0/7 (0) | 0/5 (0) |
| Rhinitis | 1/9 (0.11) | 0/11 (0) | 0/7 (0) | 0/5 (0) |
| Salpingitis | 0/9 (0) | 1/11 (0.09) | 0/7 (0) | 0/5 (0) |
| Scoliosis | 0/9 (0) | 1/11 (0.09) | 0/7 (0) | 0/5 (0) |
| Sjogren syndrome | 1/9 (0.11) | 0/11 (0) | 0/7 (0) | 0/5 (0) |
| Skin patches | 1/9 (0.11) | 0/11 (0) | 0/7 (0) | 0/5 (0) |
| Sleep apnea | 0/9 (0) | 1/11 (0.09) | 0/7 (0) | 0/5 (0) |
| Type 2 diabetes | 0/9 (0) | 1/11 (0.09) | 0/7 (0) | 0/5 (0) |
| Viral meningitis | 0/9 (0) | 1/11 (0.09) | 0/7 (0) | 0/5 (0) |
| Viral pancreatitis | 1/9 (0.11) | 0/11 (0) | 0/7 (0) | 0/5 (0) |
| Viral thyroiditis | 0/9 (0) | 1/11 (0.09) | 0/7 (0) | 0/5 (0) |

Presentation of different characteristics of the four clusters of ME/CFS patients. The results are shown in years for age and illness duration and in hours for daily activities. All data are represented as mean ± standard error of the mean. ANOVA was used to consider differences, and the results were considered significant at *P*-value ˂ 0.05 (*).
